# Eggs to long-legs: embryonic staging of the harvestman *Phalangium opilio* (Opiliones), an emerging model arachnid

**DOI:** 10.1186/s12983-022-00454-z

**Published:** 2022-03-04

**Authors:** Guilherme Gainett, Audrey R. Crawford, Benjamin C. Klementz, Calvin So, Caitlin M. Baker, Emily V. W. Setton, Prashant P. Sharma

**Affiliations:** Department of Integrative Biology, University of Wisconsin-Madison, 438 Birge Hall, 430 Lincoln Drive, Madison, WI 53706 USA

**Keywords:** Development, Chelicerata, Evo-devo, *vasa*, *engrailed*, Eupnoi

## Abstract

**Background:**

The comparative embryology of Chelicerata has greatly advanced in recent years with the integration of classical studies and genetics, prominently spearheaded by developmental genetic works in spiders. Nonetheless, the understanding of the evolution of development and polarization of embryological characters in Chelicerata is presently limited, as few non-spider species have been well studied. A promising focal species for chelicerate evo-devo is the daddy-long-legs (harvestman) *Phalangium opilio*, a member of the order Opiliones. *Phalangium opilio*, breeds prolifically and is easily accessible in many parts of the world, as well as tractable in a laboratory setting. Resources for this species include developmental transcriptomes, a draft genome, and protocols for RNA interference, but a modern staging system is critically missing for this emerging model system.

**Results:**

We present a staging system of *P. opilio* embryogenesis that spans the most important morphogenetic events with respect to segment formation, appendage elongation and head development. Using time-lapse imaging, confocal microscopy, colorimetric in situ hybridization, and immunohistochemistry, we tracked the development of synchronous clutches from egg laying to adulthood. We describe key events in segmentation, myogenesis, neurogenesis, and germ cell formation.

**Conclusion:**

Considering the phylogenetic position of Opiliones and the unduplicated condition of its genome (in contrast to groups like spiders and scorpions), this species is poised to serve as a linchpin for comparative studies in arthropod development and genome evolution. The staging system presented herein provides a valuable reference for *P*. *opilio* that we anticipate being useful to the arthropod evo-devo community, with the goal of revitalizing research in the comparative development of non-spider arachnids.

**Supplementary Information:**

The online version contains supplementary material available at 10.1186/s12983-022-00454-z.

## Background

Comparative studies with rich taxonomic sampling are essential to understanding the evolution of development. For instance, in arthropods, the molecular mechanisms of simultaneous formation of segments in the fruit fly *Drosophila melanogaster* by means of gap, pair-rule, and segment polarity genes was initially established as a paradigm of arthropod segmentation [1]. Subsequent investigation of alternative model systems across different arthropod groups later supported the hypothesis that sequential addition of segments mediated by Notch signaling is the ancestral mode of segmentation in Arthropoda [1, 2]. The discovery of a vertebrate-like oscillatory mechanism of posterior segment addition in an array of non-dipteran arthropods ignited fierce debate over the homology of segmentation in Bilateria [3–6]. Key to the phylogenetic polarization of character states within the phylum Arthropoda, and specifically, at the root of the phylum, is Chelicerata, the sister group to the remaining arthropods.

Studies on the embryology of Chelicerata flourished in the first half of the twentieth century, with classical descriptive and experimental works that have lain the foundations for the evolution of development in this group (reviewed by [7, 8]). Unification of chelicerate embryology with developmental genetics has, however, accelerated just in the last two decades [9–20]. These studies, mostly centered on two focal spider species, have led to a renaissance in the field of chelicerate development and revealed novel aspects of their evolutionary history. As an example, a modern reinvestigation of the classic graft experiment of axis specification in spiders [21] identified a genetic mechanism shared with insect dorso-ventral axis patterning (i.e., *decapentaplegic* signaling) as the basis for the activity of the cumulus, an axis organizer in spiders [9, 10, 22, 23].

Nevertheless, the embryology of non-spider arachnids has received considerably less attention in the recent literature. Orders such Pseudoscorpiones, Thelyphonida (vinegaroons), and Amblypygi (whip-spiders) suffered from decades of hiatus in contributions to their embryology (but see [24] and [25]). Embryogenesis in other orders, such as Ricinulei (hooded tick-spiders), Palpigradi (microwhip scorpions), and Solifugae (camel spiders), remains virtually unknown [7, 26]. A modern understanding of the genetic mechanisms underlying the development of non-spider arachnids has great potential to elicit evidence for major evolutionary transitions within Chelicerata, including the parallel evolution of aerial respiratory structures and the specification of different types of appendages [26, 27]).

One arachnid species that has emerged in the evo-devo literature as a focal taxon for comparative studies is the daddy-long-legs *Phalangium opilio* Linnaeus 1758 [27–39], a member of the order Opiliones (commonly known as “harvestmen”), and the first species of Opiliones described with the Linnean system. Opiliones is the third-largest order of Arachnida (sensu [40]; arachnids likely include horseshoe crabs), with over 6,500 described species [41, 42]. Among the remarkable features of this group are unique repugnatorial glands for deterring predators [43], multiple origins of parental care, and highly diverse, sexually dimorphic armature [44, 45], as well as an elongated pair of legs that are modified to serve a sensory function [46–48]. The embryology of a few Opiliones species was described in detail based on light microscopy and illustrations by studies published in the twentieth century [49–54] (for historical review, see [55]) The development of *P. opilio* was first characterized by Moritz [52] but references to embryonic stages in recent studies on *P*. *opilio* development (e.g., [29, 34]) are based on the staging system provided by Juberthie [50] for the phalangiid species *Odiellus gallicus*. Beyond this discrepancy in taxonomy, a description and staging of the development of any Opiliones species using modern approaches is still missing. Such description would aid in comparisons with the better-studied spider systems, namely *Parasteatoda tepidariorum* and *Cupiennius salei* [56, 57], and serve as a resource for functional works on Opiliones developmental genetics.

Herein, we present a staging system of *P. opilio* embryogenesis using confocal microscopy, in situ hybridization, and time-lapse imaging. We tracked the development of synchronous clutches from egg laying to adulthood and described key events in neurogenesis, myogenesis, and segmentation of the body and appendages.

## Results

We aimed to maximize compatibility between the stage numbers we propose here for *P. opilio* with the detailed description of Juberthie [50] in *O. gallicus*, and particularly the descriptions of *P. opilio* embryos by Moritz [52] and Winkler [58]. For defining stages, we combined both the characteristics from the external morphology that may be observed through the vitelline membrane, as well as specific features more clearly visible in dissected embryos using fluorescent microscopy. We used both molecular and morphological markers to characterize the germ cells and describe their dynamics. We optimized antibody staining protocols for *P. opilio* and complemented the description of some of the reference stages with cross-reactive antibody staining for muscle cells (Tm1; tropomyosin) and neural cells (acetylated α-tubulin).

### Overview of development

**Fig. 1:**
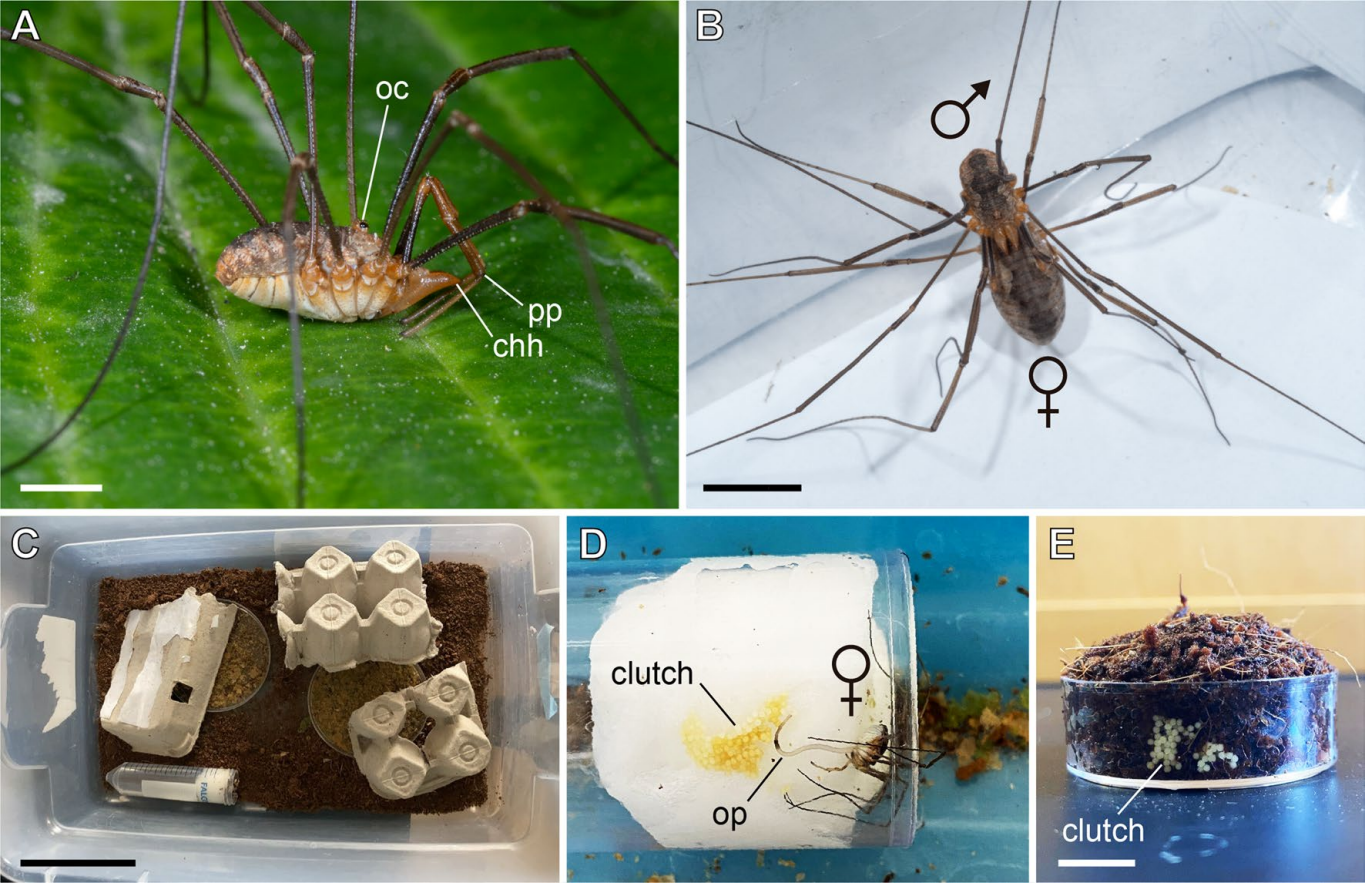
*Phalangium opilio* as a model for developmental biology. **A**: Adult male *P. opilio* in lateral view. Anterior is to the right. **B**: Mating couple of *P. opilio*. **C**: Example of a terraria where adults *P. opilio* are kept at the Sharma lab (UW-Madison). **D**: Adult female laying an egg in a clutch in dampened cotton (water source). **E**: Egg-laying dish, consisting of a 35 mm plastic dish with dampened coconut fiber. Note a clutch on the lateral wall of the dish. chh: cheliceral horn; oc: ocularium; op: ovipositor; pp: pedipalp. Scale bar (approximate values): **A**: 2 mm; **B**: 6 mm; **C**: 10 cm; **E**: 1 cm

Females of *P. opilio* lay one egg clutch approximately every three days, when provided with fresh food and a container with moist coconut fiber at 26 °C (Fig. 1C–E; Additional file 1: File S1). Upon pairing, males and females usually readily mate after a few seconds (Fig. 1B). Adult animals can be collected in temperate regions of North America, Europe, East Asia, as well as New Zealand. In north America (data available from Massachusetts and Wisconsin), adults are found in early June and the breeding season ends around early September. Nonetheless, we can successfully maintain overwintering colonies in the laboratory, and new adult females are able to breed and lay eggs during winter months. The adult lifespan is 40–60 days.

A typical egg clutch contains approximately 250 embryos (Fig. 1D–E) that develop nearly synchronously. Eggs measure approximately 550 µm in diameter, are light-yellow in color, and are almost spherical. The egg has a thin, opaque chorion and a thick, transparent vitelline membrane. The chorion is easily removed with 100% commercial bleach for fixations (see Methods). Most embryos dechorionated with 50% bleach solution can continue development and hatch in halocarbon oil or 1 × phosphate buffered saline (PBS). Upon laying, embryos from this population require around 24 days (582 h, clutch #4) to hatch at 26 °C. A summary figure of all embryonic stages, diagnostic features and timing is provided in Additional file 2: File S2.

### Embryonic staging

- Stages 1–3: Initial cleavages (days 1–4)

**Fig. 2:**
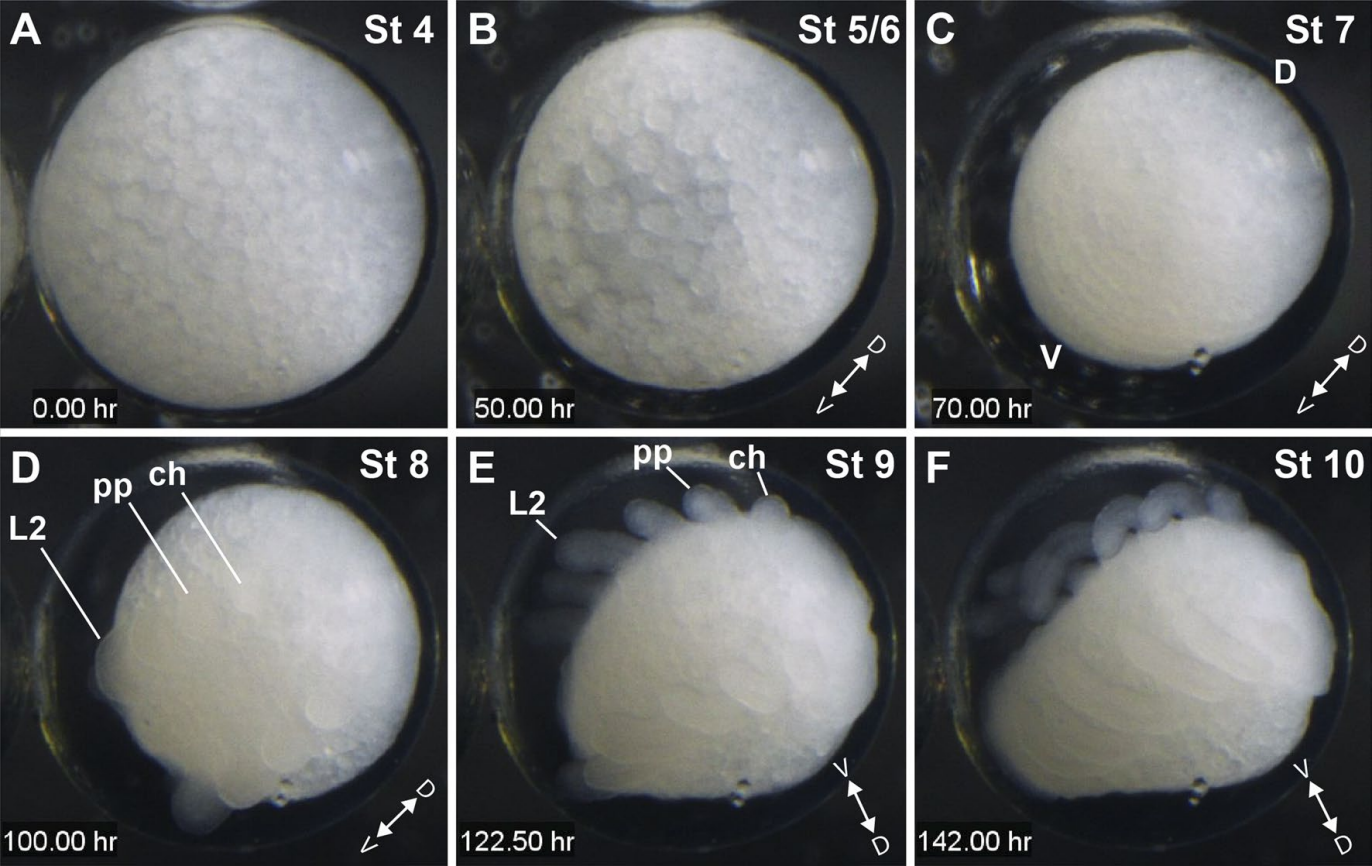
Series of selected frames from time-lapse 1 (Additional file 3: File S3) of an embryo imaged under halocarbon oil at room temperature (20 °C). Photographs were taken every 15 min. **A**: Stage 4, three-day old embryo, at the onset of perivitelline space formation. **B**: Stage 5/6, undergoing cell movements. **C**: Stage 7, with a ventral segmented germ band. **D**: Stage 8, limb bud protrusion. **E**: Stage 9, appendage elongation and further formation of opisthosomal segments. **F**: Stage 10: Ventral inflexion of the opisthosoma. The diameter of the egg is approximately 550 µm. Timing of development is slower than the staging given the cooler temperature at which the embryo was imaged. D and V: dorso-ventral axis; ch: cheliceral segment; L2: leg 2 segment; pp: pedipalpal segment

Stages encompassing the first cleavages and a blastoderm with loosely arranged yolk globules are here collectively treated as stages 1–3 until further study (see Discussion), following Fig. 20 of Juberthie [50] in the description of *Odiellus gallicus*. Embryos in this range proved challenging to investigate in *P. opilio*. In very early embryos, yolk cells and embryonic cells present a similar coloration and thus the initial cleavages were not observed at these early stages through the vitelline membrane. Attempts to manually remove the vitelline membrane in fixed embryos to observe cells at stages before the blastoderm formation resulted in the disintegration of the embryo, suggesting that the cells have limited cohesion at this stage, even after extended fixation periods. The broad range of autofluorescence of this membrane hampers visualization of cells under a fluorescent stereomicroscope with Hoechst and GFP. From egg laying to 72 h after egg laying (hAEL) (stages 1–3), we observed loosely arranged, large yolk globules at the periphery of the embryo (Fig. 2A). We also observed the overall dynamic of the cells in these early three stages by performing time-lapse imaging of 3-day-old embryos. Before the formation of the perivitelline space (see below), we observed movement of the yolk globules (Additional file 3: File S3).

(b) Stages 4–6: Late blastoderm and germ band (day 5; 120–147 hAEL)

#### Stage 4: Late blastoderm

**Fig. 3:**
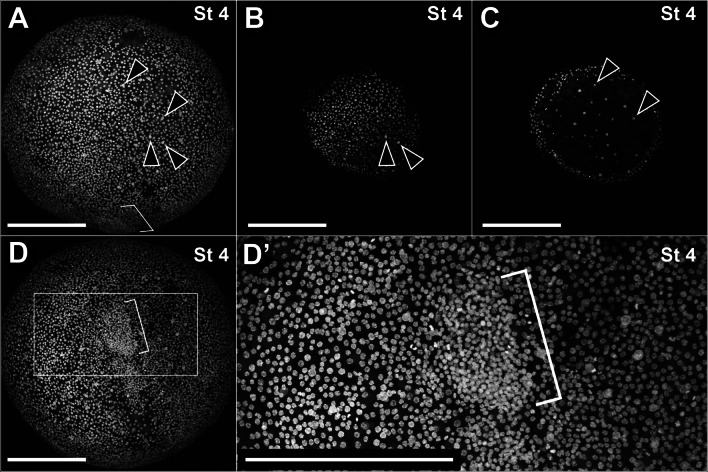
Stage 4 (**A**–**D**′). Confocal micrographs of an embryo of *P. opilio* stained with Hoechst (nuclei). **A**: Z-stack maximum intensity projection of the whole embryo. **B**, **C**: Optical sections at 6 and 11 µm depths (outward to inward), respectively. Note that larger nuclei occur more internally to the small nuclei. **D**: Z-stack maximum intensity projection of surface of the embryo shown in “**A**” rotated 90° forward. **D**′: detail of the same image in **D**, showing a denser spot of small nuclei at the surface of the embryo (bracket). Arrowheads: larger sub-peripheral nuclei. Scale bar **A**–**D**′: 200 µm

A stage 4 embryo is diagnosed by the first sign of a perivitelline space. When approaching 120 h (5 days), the embryo cells look more cohesive and the perivitelline space appears. (Fig. 2A; Additional file 3, 4: File S3–S4). Fixed embryos at this stage may be peeled from the vitelline membrane and observed under a fluorescent stereomicroscope. A stage 4 embryo is nearly spherical and has one layer of cells with small nuclei surrounding the yolk globules (Fig. 3A, D). A few larger nuclei are present immediately below the outer layer of small nuclei and they are roughly regularly spaced (Fig. 3A–C). A denser elliptical spot of multilayered cells is observed (Fig. 3D, D′).

#### Stage 5: Early germ band (130 hAEL)

**Fig. 4:**
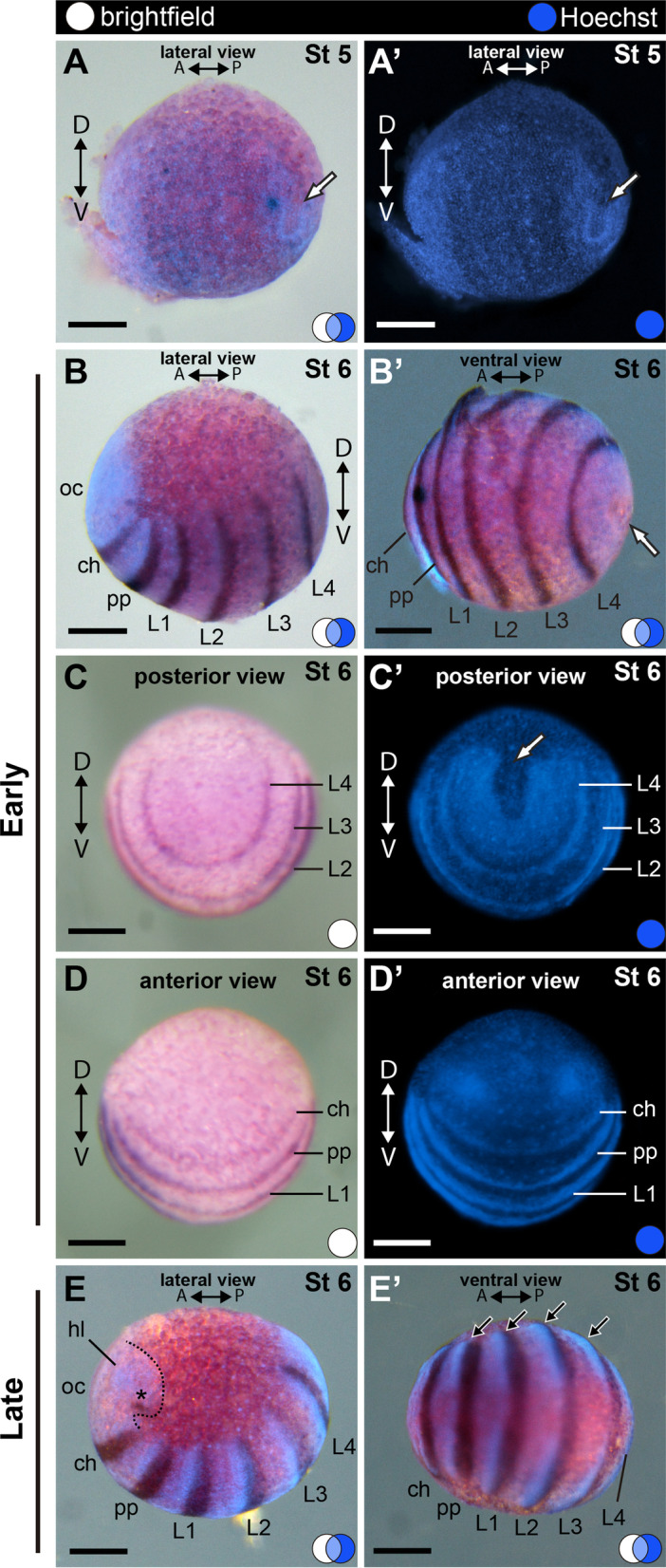
Stage 5 (**A**) and early (**B**–**D**′) to late (**E**–**E**′) stage 6. In situ hybridization for *Popi-en*. **A**, **A**′: Early germ band. No clear expression of *Popi-en* was detected. **B**–**D**′: Initial segmentation of the prosoma. **D**–**E**′: C-shaped segmented germ band longer in the A–P axis and with more defined head-lobe. A and P: anterior–posterior axis; asterisk: dot of *engrailed* expression; D and V: dorsal–ventral axis; white arrow: horseshoe shaped posterior end of the germ band; blue circle: Hoechst staining. white circle: bright field; ch: cheliceral segment; L1–4: leg 1–4 segments; oc: ocular segment; pp: pedipalpal segment. Scale bars: 100 µm

Stage 5 is defined by the formation of a horseshoe-shaped region on the blastoderm (Fig. 2B; Fig. 4A, A′; white arrow) and expansion of the perivitelline space (Fig. 2B; Additional file 3, 4: File S3–S4). The germ band is recognizable as a denser layer of cells on a hemisphere of the yolk, being the ventral side of the embryo, with an antero-posterior axis; the posterior end assumes a horseshoe shape (Fig. 4A, A′). At this stage, a whiter group of slightly elevated cells occurs at the posterior of the embryo (Additional file 4: File S4, frame 100 h, yellow arrowhead). The anterior end has a denser layer of cells, at the place of the future cephalic region. No clear expression of the segment marker *Popi-engrailed* (*Popi-en*) could be detected by in situ hybridization at this stage (Fig. 4A, A′).

#### Stage 6: segmented germ band (140 hAEL)

**Fig. 5:**
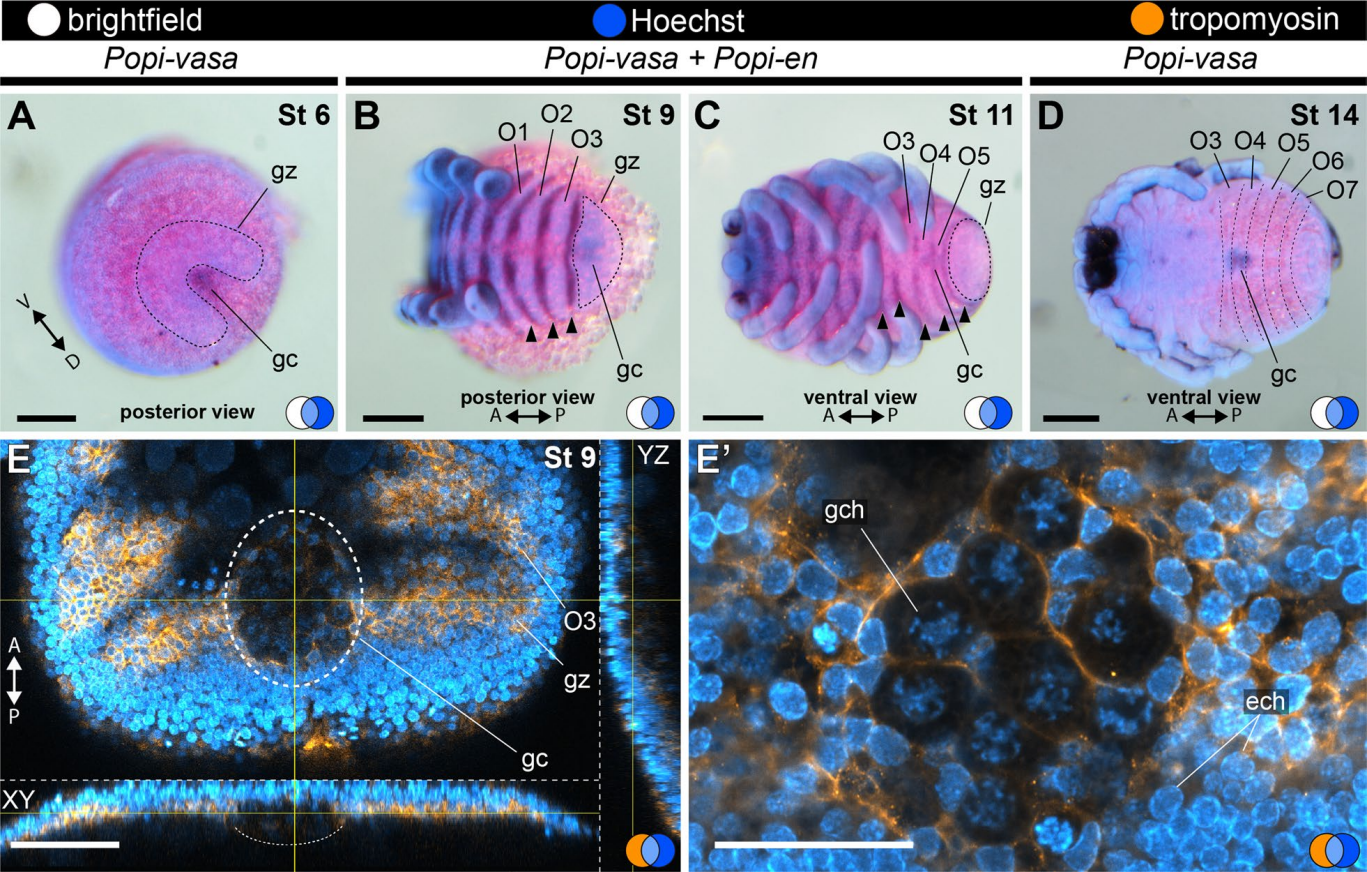
Germ cell development in *P. opilio*. **A**, **D**: In situ hybridization for *Popi-vasa*. **B**–**C**: Double in situ hybridization for *Popi-vasa* and *Popi-en*. **E**–**E**′: Antibody staining for tropomyosin. **A**: Stage 6 embryo. **B**: Stage 9 embryo. **C**: Stage 11 embryo. **D**: Stage 14 embryo. **E**: Single optical slice through the germ cell cluster at stage 9, with orthogonal projections. **E′**: Detail of the germ cell nuclei shown in “**E**”. A and P: antero-posterior axis; blue circle: Hoechst; orange circle: tropomyosin; white circle: bright field; ech: ectodermal cell chromatin; gc: germ cell cluster; gch: germ cell chromatin; gz: growth zone; O3–O7: opisthosomal segments 3–7. Scale bar **A**–**D**: 100 µm; **E**: 50 µm; **E**′: 25 µm

At stage 6 the germ band is narrower than in stage 5 and has seven prosomal segments (i.e., the anterior tagma of chelicerates) and a posterior growth zone (Fig. 4B–E′). Stage 6 is therefore marked by the segmentation of the prosoma. The ocular segment (the most anterior) expresses *Popi-en* as a dot (Fig. 4E; asterisk). The other six segments express *Popi-en* as a stripe at their posterior edge (a conserved expression domain in Arthropoda [59] (Fig. 4B–E′). Early stage 6 is nearly spherical and the *Popi-en* stripes of expression are thin and uniform across the left and right edges of the germ band (Fig. 4B–D′). The horseshoe-shaped posterior germ band is sharply defined as a segment growth zone (Fig. 4C, C′; white arrow). The whiter spot of cells is surrounded by the horseshoe-shaped growth zone, posterior to it (Fig. 5A). These cells express the germ cell marker *Popi-vasa* throughout embryogenesis (Fig. 5A–D).

Primordia of the head lobes are formed, initially appearing as two dense spots (Fig. 4D, D′). At a late stage 6, the germ band extends posteriorly as each segment becomes broader, and the embryo assumes a more oval shape. Each prosomal segment becomes denser where the limb buds will protrude (Fig. 4E′; black arrows). The head lobes expand laterally at the anterior end of the germ band (Fig. 4E; dotted line). The stage numbers from this point up to stage 12 match the number of *engrailed* stripes posterior to the ocular segment. The convention of stages using segments as landmarks is particularly useful for studying *P. opilio* embryogenesis, because stages 6–13 encompass appendage formation and elongation, segmentation of the body, and head elaboration.

(iii) Stages 7–14: elaboration of the head and formation of the opisthosoma (days 6–10; 147–258 hAEL)

#### Stage 7: first opisthosomal segment (147 hAEL)

**Fig. 6:**
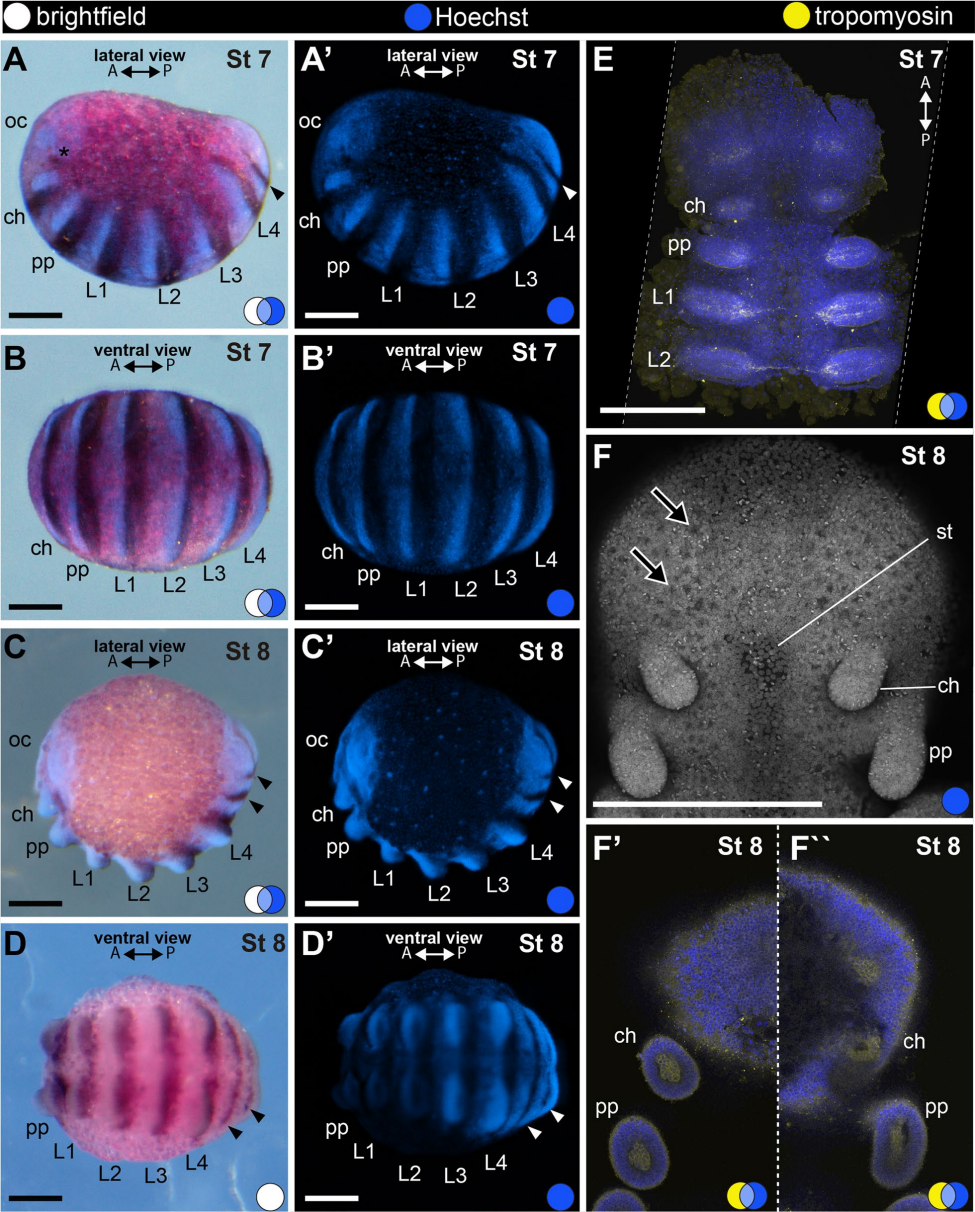
Stage 7 (**A**–**B**′, **E**) and stage 8 (**C**–**D**′, **F**–**F**′′). In situ hybridization for the gene *Popi-engrailed* (**A**–**D**′) and confocal micrographs of fluorescent antibody staining against tropomyosin (**E**–**F**′′). Images with the same letters are different views of the same embryo. **A–B**′: First opisthosomal segment. **C**–**D**′: Second opisthosomal segment. **E**: Anterior half of a flat-mounted germ band (ventral view). Tropomyosin-positive cells occur at the head lobe and primordial limb buds. **F**: Anterior third of a flat-mounted germ band at stage 8 (ventral view). Stomodeum begins to form between cheliceral limb buds. **F**′, **F**′′: Optical sections at, respectively, 16 and 27 µm depth from the ventral surface of the same sample in **F**, showing tropomyosin-positive cells internal to each limb bud, and a coelomic space. A and P: anterior–posterior axis; arrowhead: stripe of *engrailed* expression marking the posterior border of opisthosomal segment; asterisk: dot of *engrailed* expression; black arrow: invagination site of neural precursor cells; blue circle: Hoechst (blue); white circle: bright field; yellow circle: tropomyosin (yellow); ch: cheliceral segment; L1–4: leg 1–4 segments; oc: ocular segment; pp: pedipalpal segment; st: stomodeum. Scale bars **A**–**D**′: 100 µm. **E**–**F**′′: 250 µm

Stage 7 is defined by the occurrence of the first opisthosomal segment, and the oblong shape of the embryo (Fig. 6). From this stage onwards, each of the nine opisthosomal segments form sequentially from the anterior border of the posterior growth zone. Each new segment expresses *Popi-en* at its posterior border (Fig. 6A–B). Stage 7 also encompass the initial formation of the prosomal limb buds (chelicera, pedipalp, L1-L4 legs), which slightly bulge from the germ band and are wider than longer (Fig. 6A, B, E). The expression of *Popi-en* concentrates on the lateral edges of each prosomal segment and appears fainter at the ventral midline of the embryo (Fig. 6A–B).

#### Stage 8: second opisthosomal segment, limb buds (153 hAEL)

Stage 8 has two opisthosomal segments formed and limb bud primordia with a proximo-distal axis (longer than wider) (Fig. 2D; Fig. 6C–D', F–F'). The limb bud of the second pair of legs is slightly longer than the other appendages (Fig. 6C–D', F–F') and this allometry is maintained from this stage to adulthood, when this elongated leg assumes a sensory function. The primordium of the stomodeum begins to form medially on the head lobes (Fig. 6F). The germband is narrower than in the previous stage (compare Fig. 6B and D). Each limb bud is composed of an external layer of 3–5 cells that do not stain for tropomyosin, and an internal layer of cells staining for tropomyosin (Fig. 6F′, F′′). A space exists between these two layers of cells, and the growing limb buds maintain this space along the appendages as they elongate, so that the bundle of cells stained for tropomyosin traverses the tube-like appendage (Fig. 6F′, F′′).

#### Stage 9: third opisthosomal segment (171 hAEL)

**Fig. 7:**
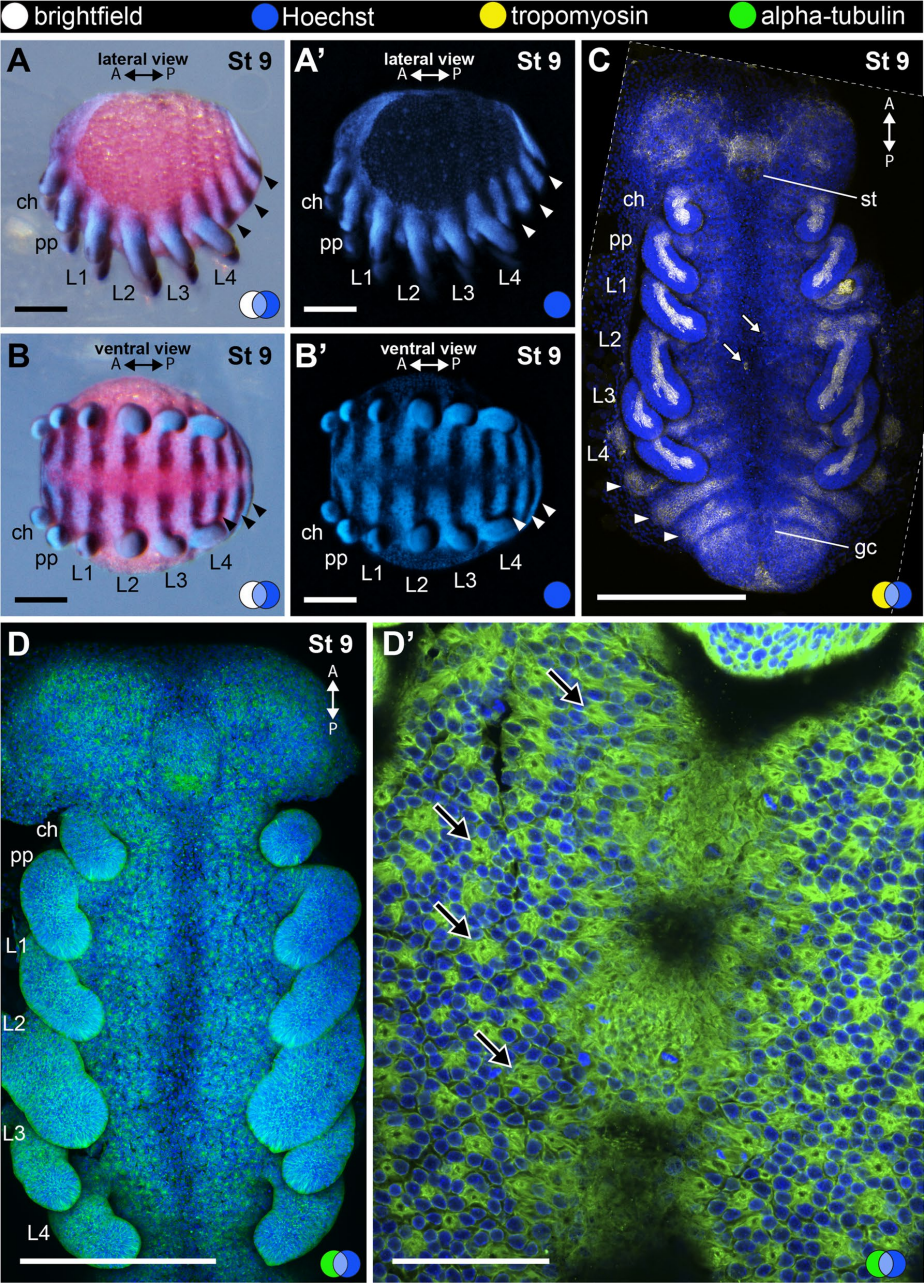
Stage 9 (**A**–**D**′). In situ hybridization for the gene *Popi-engrailed* (**A**–**B**′) and confocal micrographs of fluorescent antibody staining against tropomyosin (**C**) and alpha-tubulin (**D**, **D**′). Images with the same letters are different views of the same embryo. **A**–**B**′: Third opisthosomal segment. **C**: Maximum projection of a flat-mounted embryo (ventral view). Tropomyosin-positive cells occur as a bundle inside each prosomal limb buds and in broad stripes in each opisthosomal segment and posterior growth zone. **D**: Maximum projection of a flat-mounted embryo (ventral view). Note groups of invaginating cells along ventral ectoderm and head lobes. **D**′: Detail of a group of invaginating neural precursor cells on the ventral ectoderm. A and P: anterior–posterior axis; arrowhead: stripe of engrailed expression marking the posterior border of opisthosomal segment; black arrow: neural precursor cells (apical process); blue circle: Hoechst (blue); white arrow: isolated tropomyosin-positive cell; white circle: bright field; yellow circle: tropomyosin (yellow); green circle: alpha-tubulin (green); ch: cheliceral segment; gc: germ cell cluster; L1–4: leg 1–4 segments; pp: pedipalpal segment; st: stomodeum. Scale bars **A**–**B**′: 100 µm. **C**, **E**: 250 µm. D reprinted with permission from Sharma (2018) Current Biology 28, R774–R778

Stage 9 is defined by the presence of three opisthosomal segments (Fig. 7A–C). The horizontal stripes of *Po-en* are interrupted along the developed ventral sulcus (Fig. 7B, B′). The labrum has begun to form, but does not yet project posteriorly to cover the stomodeum. Cells expressing tropomyosin are present on the head lobes and on the developing labrum anterior to the stomodeum, which now is open (Fig. 7C). Cells expressing tropomyosin also occur as two bilaterally symmetrical horizontal bands at each body segment, below the outer layer of ectodermal cells and interrupted at the ventral sulcus (Fig. 7C). As the development of legs progresses, tropomyosin-positive cells form a continuous bundle from the base to the tip of each appendage. At late stage 8 (Fig. 6; black arrows) and more clearly from stage 9 onwards, neural precursor cells differentiate and invaginate in the ventral ectoderm and head lobes. The apical process of invaginating cells appears as a bright spot of alpha-tubulin expression, which is enriched in neural cells (Fig. 7D–D′; black arrows). The sites of invagination are regularly spaced in the ventral ectoderm and are surrounded by 6–8 ectodermal cells, forming rosettes (Fig. 7D–D′; black arrows).

We further analysed the germ cells at stage 9. The germ cells are distinctively larger than surrounding cells (Fig. 5E) and occur below the ectoderm (Fig. 5E). The nuclei of the germ cells have loosely compacted chromatin compared to the ectodermal cells (Fig. 5E′). The germ cell cluster at stage 9 occurs anterior to the growth zone and posterior to O3 (in contrast to posterior to the growth zone in stage 6). By the end of stage 9, the germ cell cluster is localized in the nascent O4 (Fig. 5B, E). From stage 9, until at least stage 14, the germ cell cluster remains within O4 and O5, as evidenced by the co-visualization of *Popi-en* and *Popi-vasa* (Fig. 5).

#### Stage 10: fourth opisthosomal segment (174 hAEL)

**Fig. 8:**
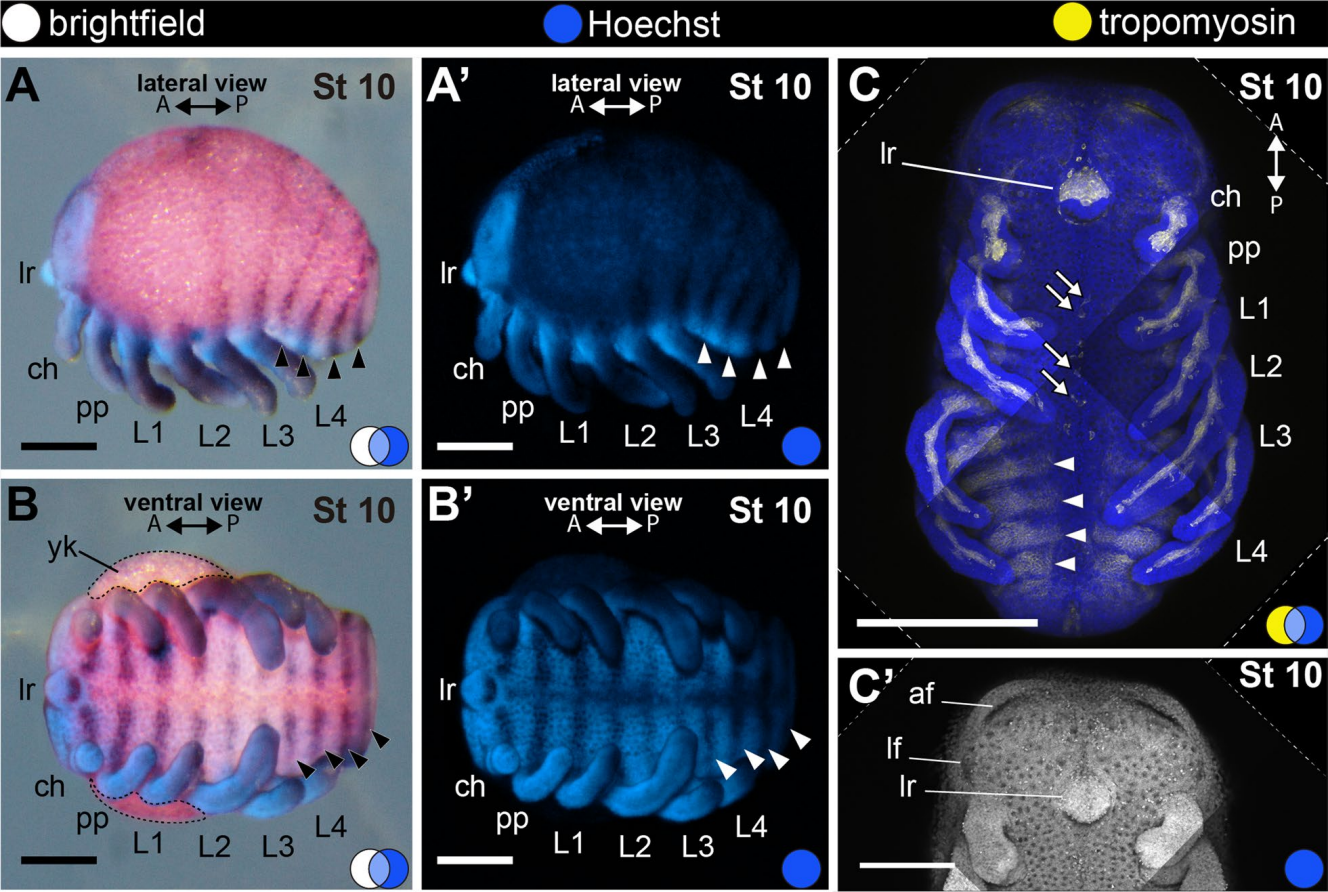
Stage 10 (**A**–**C**′). In situ hybridization for the gene *Popi-engrailed* (**A**–**B**′) and confocal micrographs of fluorescent antibody staining against tropomyosin (**C**, **C**′). **A**–**B**′: Fourth opisthosomal segment and ventral flexure of the opisthosoma. **C**: Maximum projection of a flat-mounted embryo (ventral view). **C**′: Detail of the head lobes of same preparation shown in **C**. Only Hoechst channel in shown (gray). A and P: anterior–posterior axis; arrowhead: stripe of *engrailed* expression marking the posterior border of opisthosomal segment; blue circle: Hoechst (blue, gray); white arrow: isolated tropomyosin-positive cell; white circle: bright field; yellow circle: tropomyosin (yellow); af: anterior furrow; ch: cheliceral segment; lr: labrum; lf: lateral furrow; L1–4: leg 1–4 segments; pp: pedipalpal segment; yk: yolk. Scale bars A–B′: 100 µm. C: 250 µm. C′: 125 µm

Stage 10 has four opisthosomal segments formed. The embryo is no longer C-shaped in lateral view and the opisthosoma begins to project ventrally (Fig. 2F; Fig. 8A–C). This ventral flexure may be observed in the time-lapse of developing embryos (Fig. 2F; Additional file 3: File S3). In ventral view, the yolk is visible only in the anterior half of the embryo (Fig. 8B). The bundle of tropomyosin-positive cells of the chelicera begins to branch, and externally the distal part of the appendage has a triangular shape, or a slight indentation, at late stage 10 (Fig. 8C–C′). A line of 10 cells positive for tropomyosin expression occur along the ventral sulcus spanning the prosomal segments 3–6 (Fig. 8C; white arrows). A few cells already appear in stage 9 (Fig. 7C; white arrows). We did not follow the fate of these cells further in development. The anterior and ectal rims of the head lobes begin to form the anterior furrow (cerebral fold sensu [49]; semilunar grooves, sensu [7]), and a lateral furrow (Fig. 8C; see also stage 11). The labrum resembles a “nose”, pointing posteriorly and covering the mouth in frontal view (Fig. 8A–C).

#### Stage 11: fifth opisthosomal segment (177 hAEL)

**Fig. 9:**
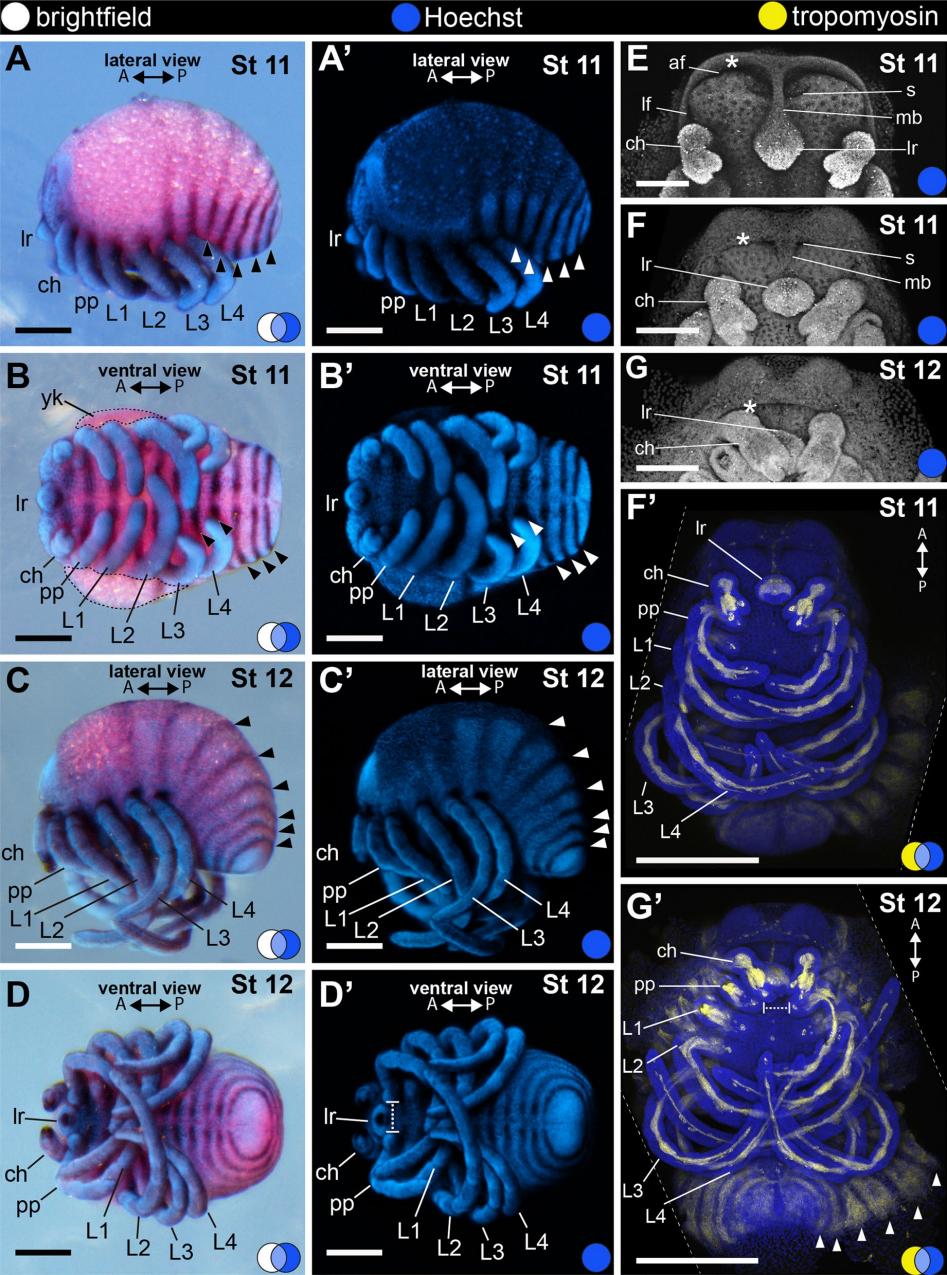
Stage 11 (**A**–**B**′, **E**–**F**′) and stage 12 (**C**–**D**′, **G**–**G**′). In situ hybridization for *Popi-en* (**A**–**D**′) and confocal micrographs of fluorescent antibody staining against tropomyosin (**F**′, **G**′). **A**–**B**′: Fifth opisthosomal segment and initial folding of the anterior furrow. **C**–**D**′: Sixth opisthosomal segment. **E**: Detail of the head lobes of early stage 11. **F**: Detail of the head lobes of late stage 11 embryo. **F**′: Maximum projection of a flat-mounted late stage 11 embryo (ventral view). **G**: Detail of the head lobes of a stage 12 embryo. **G**′: Maximum projection of a flat-mounted embryo at stage 12 (ventral view). A and P: anterior–posterior axis; arrowhead: stripe of *engrailed* expression marking the posterior border of opisthosomal segment; blue circle: Hoechst (blue, gray); white circle: bright field; yellow circle: tropomyosin (yellow); asterisk: anterior rim of the folding ectoderm; af: anterior furrow; ch: cheliceral segment; lr: labrum; lf: lateral furrow; L1–4: leg 1–4 segments; mb: medial bridge; pp: pedipalpal segment; s: slit; yk: yolk. Scale bars **A**–**G**: 100 µm. **F**′, **G**′: 250 µm

This stage is marked by the formation of the fifth opisthosomal segment. At early stage 11, L2 legs overlap crossing the ventral midline of the embryo and L3 and L4 legs are curved up (Fig. 9A–B′; 9F′). The distal podomere of the chelicera is branched (Fig. 9E–F). As in stage 10, the yolk is visible only in the anterior half of the embryo (Fig. 9B). The anterior margin of the head ectoderm moves towards the base of the chelicerae, folding over the neurogenic ectoderm of the head (Fig. 9E–G; asterisk) (completed by stage 13). At early stage 11, two slit invaginations occur adjacent to a medial bridge connecting the labrum and the anterior rim of the head (Fig. 9E). The anterior and lateral furrows deepen to surround the left and right lobes of the head, and a narrow medial bridge connects the labrum and the anterior fold (Fig. 9E). At late stage 11 the posterior margin of the folding tissue is orthogonal to the medial bridge, and the slit invaginations are still visible as two pits adjacent to the medial bridge (Fig. 9F).

#### Stage 12: sixth opisthosomal segment (216 hAEL)

Stage 12 is defined by the sixth opisthosomal segment. The anterior and lateral margins of the anterior fold cover the medial bridge and the paired slits, the latter not visible anymore in frontal view (Fig. 9C–D′, G–G′). The lateral margins of each segment expand dorsally around the yolk, so that in ventral view the dorsal yolk is not visible (Fig. 9D–D′). L2 legs cross over the germ band lateral margin on the opposite side of the embryo and curl anteriorly, while L4 legs cross the ventral midline and reach the coxae of L2 legs (Fig. 9D, D′, F). The proximal article of the chelicerae and the endites of the pedipalps do not overlap the labrum (Fig. 9D, D′, G′). The gonopores first appear at this stage flanking the ventral midline on the second opisthosomal segment (Fig. 7C, D of [34]), whereas the spiracles open more laterally on the same segment (along the same longitudinal axis as the coxa-trochanter joints of the walking legs; Fig. 8A of [34]).

#### Stage 13: seventh opisthosomal segment (228 hAEL)

**Fig. 10:**
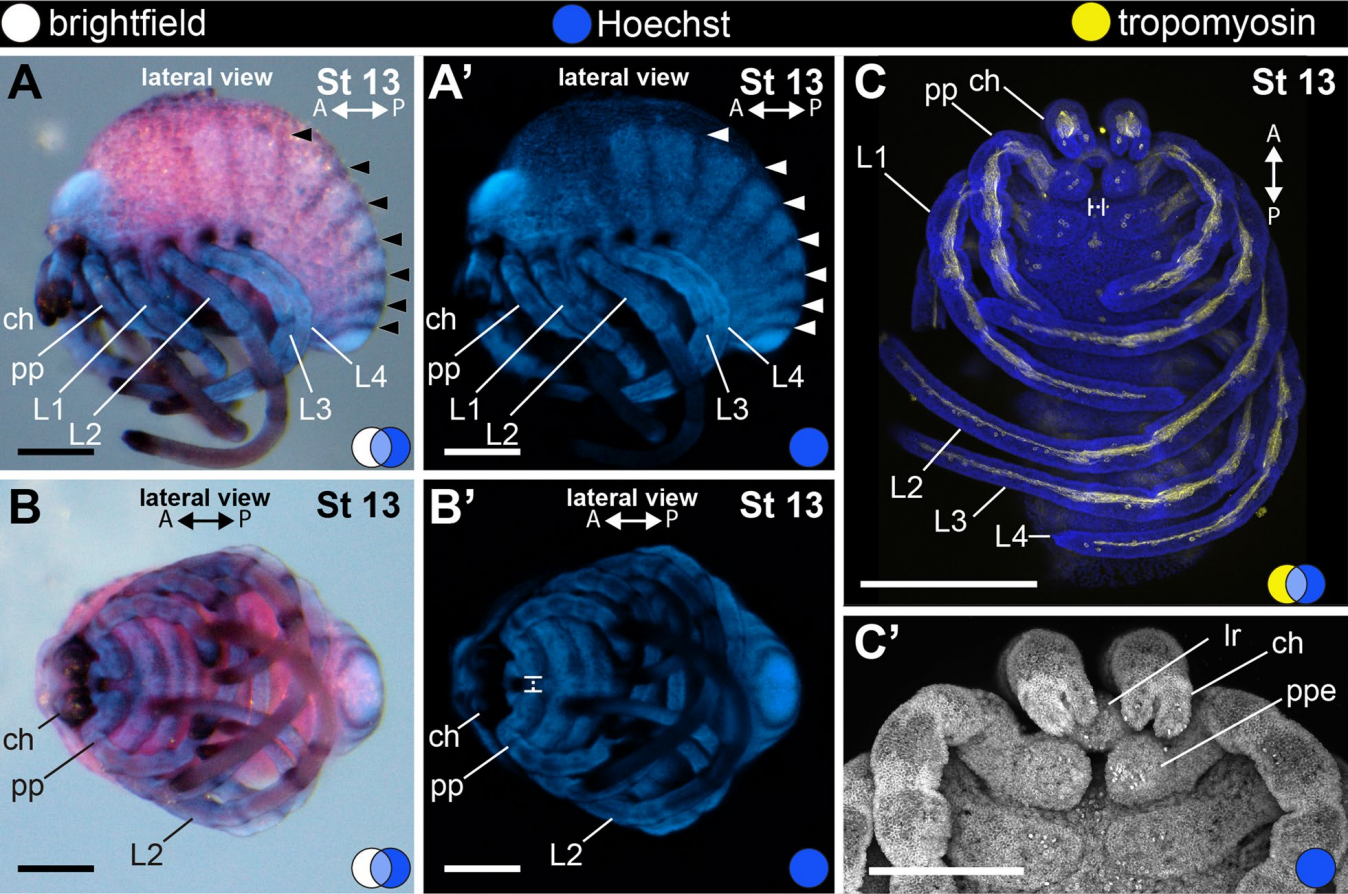
Stage 13 (**A**–**C**′). In situ hybridization for the gene *Popi-engrailed* (**A**–**B**′) and confocal micrographs of fluorescent antibody staining against tropomyosin (**C**, **C**′). **A**–**B**′: Seventh opisthosomal segment. **C**: Maximum projection of a flat-mounted embryo. **C**′: Detail of the chelicera and pedipalp (ventral view). A and P: anterior–posterior axis; arrowhead: stripe of *engrailed* expression marking the posterior border of opisthosomal segment; blue circle: Hoechst (blue, gray); white circle: bright field; yellow circle: tropomyosin (yellow); ch: cheliceral segment; lr: labrum; L1–4: leg 1–4 segments; pp: pedipalpal segment; ppe: pedipalpal endite. Scale bars A–B′: 100 µm. C: 250 µm. C′: 125 µm

Stage 13 (Fig. 10) is defined by the seventh opisthosomal segment. The anterior fold of the head has completely covered the medial bridge (Fig. 10A–B′) and forms the prosomal margin (*clypeus *sensu [49]). The proximal article of the chelicera and the endites of the pedipalps overlap with the labrum (respectively, in frontal and ventral view) (Fig. 10B–C′). Late stage 13 has parallel chelicerae and the labrum ventral to them (Fig. 10C–C′).

#### Stage 14: final opisthosomal segments (246 hAEL).

**Fig. 11:**
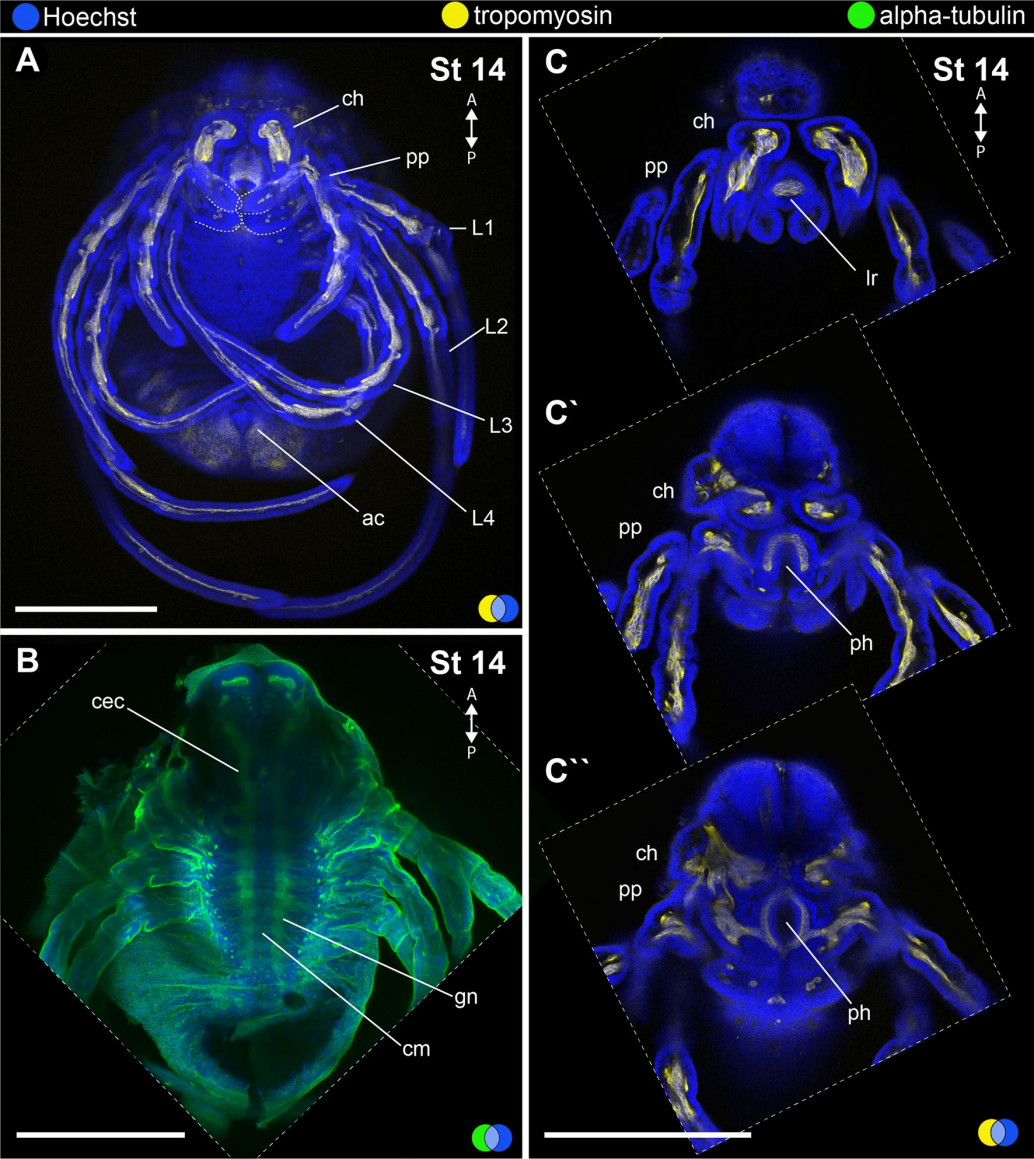
Stage 14 (**A**–**C**′′). Confocal micrographs of fluorescent antibody staining against tropomyosin (**A**, **C**–**C**′′) and alpha-tubulin (**B**). **A**: Maximum projection of flat-mounted embryo stained for tropomyosin and Hoechst (ventral view). Dotted lines mark the coxae of pedipalps and L1 legs. **B**: Maximum projection of mid-optical section of flat-mounted embryo stained for alpha-tubulin and Hoechst (ventral view). Anterior appendages have been dissected for clarity. **C**–**C**′′: Optical sections of the head region of the same preparation in **A**. Note the tubular pharynx musculature. A and P: anterior–posterior axis; blue circle: Hoechst (blue); yellow circle: tropomyosin (yellow); green circle: tropomyosin (green); ca: corona analis. ch: chelicera segment; cec: circumesophageal connective; cm: commissure; gn: ganglium; lr: labrum; L1–4: leg 1–4 segments; ph: pharynx; pp: pedipalp. Scale bars: 250 µm. B reprinted with permission from Sharma (2018) Current Biology 28, R774–R778

Stage 14 (Fig. 11) is defined by the presence of the *corona analis*, a projection of the opisthosomal end comprising the opisthosomal segments 8–9 and the telson around the proctodeum (Fig. 11A) (see also [58]). The chelicera has fixed and movable digits bearing pointed termini (Fig. 11A). The coxapophyses of the pedipalp and L1 legs meet at the ventral midline, forming the floor of the stomotheca (preoral chamber) (Fig. 11A; dotted lines). The nervous system at this stage is well developed, with marked circumpharyngeal connectives to the brain and ventral nerve cord with parallel commissures (Fig. 10B) (not analysed at previous stages).

(iv) Stages 15–19: elaboration of the eyes, organogenesis, and cuticular characters (days 10–22)

According to Holm [49] and Juberthie [50], organogenesis begins with the end of segmentation, and proceeds until the embryo hatches. Formation of the digestive caeca and the heart have been addressed in previous works (see [50, 52, 58]), and occur during stages 15–19 (see below) [50, 52, 58]. We defined the final stages based on external landmarks visible in bright field, and that are evenly spaced in time from stage 14 up until hatching.

#### Stage 15: Eye pigmentation (day 10)

**Fig. 12:**
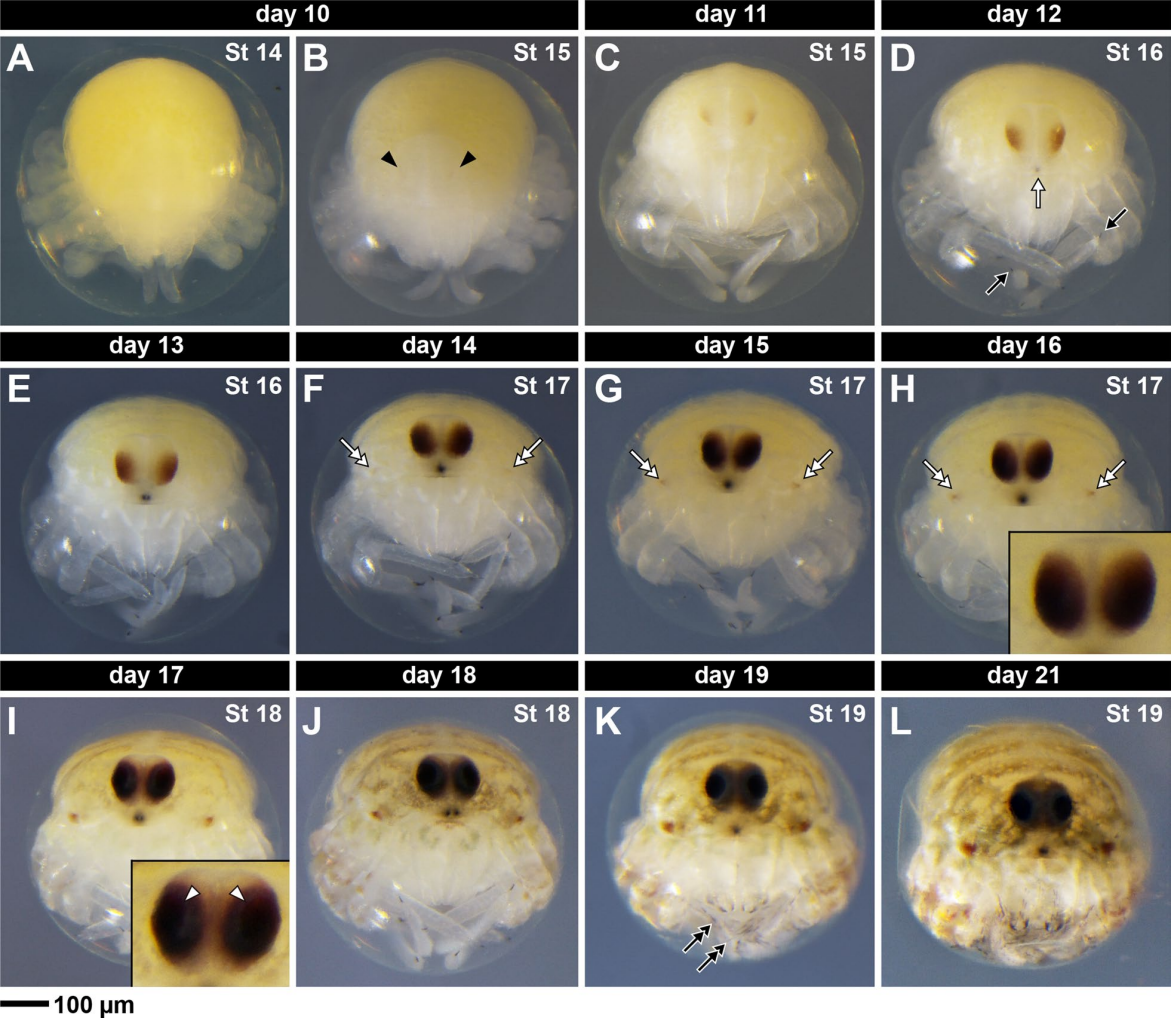
Stage 14 (**A**), stage 15 (**B**–**C**), stage 16 (**D**–**E**), stage 17 (**F**–**H**), stage 18 (**I**–**J**), and stage 19 (**K**–**L**). Frontal view of live embryos from the same clutch imaged under 1 × PBS. Black arrow: tarsal claws; black double-arrow: setae; black arrowhead: eye pigment; white arrow: egg tooth; white double-arrow: ozopore; white arrowhead: eye lens. Scale bar: 100 µm

Stage 15 is defined by the onset of red pigment deposition in the eyes (Fig. 12B–C; black arrowheads). Pigment is initially restricted to the lower half of the eye (frontal view) and is light-red colored in bright field (Fig. 12B–C).

#### Stage 16: Egg tooth pigmentation (day 12)

Stage 16 is defined by the darkening of the tarsal claws in the pedipalp and L1–L4 legs (Fig. 12D–E; black arrows), and the darkening of the egg tooth, an embryonic structure anterior to the eyes in the margin of the prosoma (Fig. 12D–E; white arrow).

#### Stage 17: Ozopore pigmentation (day 14)

Stage 17 is defined by the onset of red pigmentation around the ozopores, which are the openings of the repugnatorial glands (Fig. 12F–H; white double-arrows). It is unclear if the pigmentation derives from the gland itself, or whether it is a pigmentation of the cuticle.

#### Stage 18: Lens formation (day 17)

Stage 18 is defined by the appearance of a distinct cuticular lens in the eye (Fig. 12I–J; white arrowheads). The lens is easily discernible because the pigmentation in the eye is black in the lens area, and grey-blue colored around it (Fig. 12I–J; compare to Fig. 12E–H). Patches of pigmentation in the dorsal prosomal cuticle start to become visible at this stage, and darken up until hatching (Fig. 12I–L).

#### Stage 19: Setae and chelicera claw darkening (day 19)

This last stage before hatching is marked by the darkening of setae on the appendages, and of the movable and fixed finger of the chelicerae (Fig. 12K–L; black double-arrows). Movements of the body and appendages are clearly visible.

(e) Hatching and post-embryonic development

The term ‘larva’ has been used in the harvestman literature to describe the stage immediately after hatching [50, 55], because it lacks certain features of the next instars, such as setae. In the spider literature, the term was evaluated with some criticism by Wolff and Hilbrant [57] due to the confusing homology schema with insects implied by its use. In their staging system of the ctenid spider *C. salei*, Wolff and Hilbrant [57] proposed the alternative “postembryo” to describe the spider hatchling, which is immobile, lacks sensory hairs, and has only partially developed eyespots; only after molting from this stage does the spider become a first instar. This convention was adopted by subsequent investigations of spider development (e.g., [56]). On the same grounds, we here adopt the term postembryo to refer to the hatchlings of *P. opilio*.

**Fig. 13:**
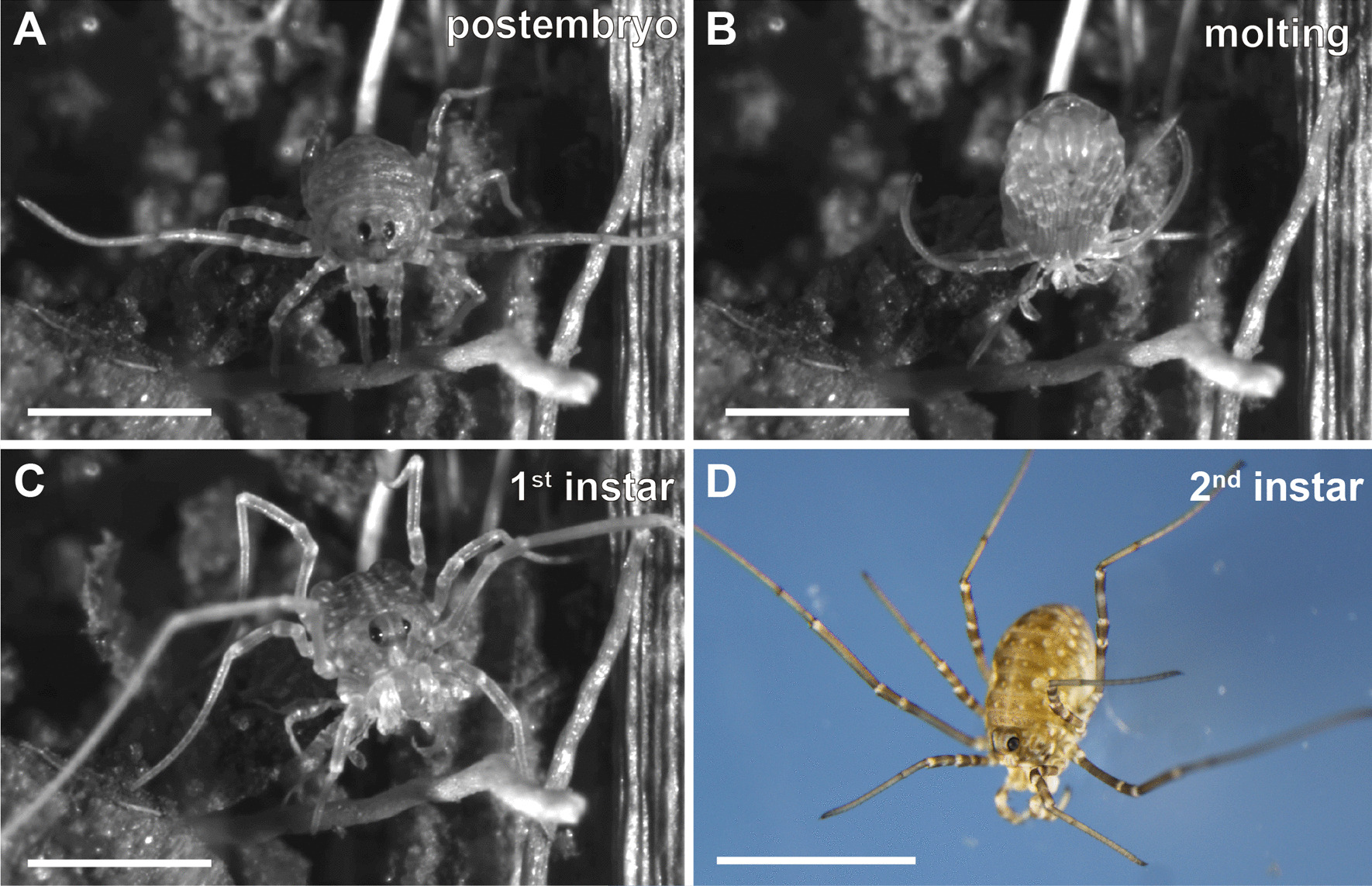
Postembryonic stages of *Phalangium opilio*. **A**–**C**: frames of a time-lapse imaging of postembryo moulting into first instar. **A**: postembryo. **B**: undergoing molting. **C**: 1^st^ instar. **D**: 2^nd^ instar. Scale bars: **A**–**D**: 1 mm

Embryos hatch into postembryos between 22 and 24 days after oviposition at 26ºC (Additional file 5: File S5). The postembryos of *P. opilio* have a flexible opisthosoma, which telescopes in and out periodically. In contrast to the spider postembryo, the hatchling of *P. opilio* is mobile, has well developed eyes, and has already produced setae under the postembryo cuticle. Within 2 h of hatching, postembryos undergo the first molt and reach the first instar stage (Fig. 13A–C; Additional file 6: File S6). This fast molting is likely possible because of precocious formation of the first instar cuticle inside the postembryo cuticle while still inside the egg (clearly visible in distal appendage termini; see Fig. 3 of [38]). The second instar is reached seven days after hatching (Fig. 13D). Six instars occur before the final molt into adulthood (Table 1), but this number may vary from 6–8 in different populations of this species [55]. Molting is approximately synchronous up to the 5th instar, when the first signs of sexual dimorphism in the male chelicera (horns) begin to appear. At the sixth instar, immature males have a more delicate body than immature females and exhibit primordia of the cheliceral horns. Of the two animals that survived to adulthood from the tracked clutch, one was a male and one a female. The male molted to adulthood at day 48 after hatching and the female at day 55 after hatching (Table 1).

**Table 1:** Duration of post embryonic instars, measured from data of a single clutch

Stage	Time after hatching
Postembryo	day 0; n ≥ 3
1st instar	day 0 (2 h); n ≥ 3
2nd instar	day 7; n ≥ 3
3rd instar	day 13; n ≥ 3
4th instar	day 23; n = 2
5th instar	day 34; n = 2
6th instar	day 43; n = 2
Adult	#1(male): day 48; #2(female): day 55

## Discussion

### Early embryogenesis and germ cells

Only a few studies have documented the early cleavages and formation of the blastoderm in Opiliones, and many aspects remain unclear. According to Holm [49], the first cleavages are believed to be intralecithal [49], which follows the general pattern of other arachnids [7]. Juberthie [50] depicted a blastoderm stage composed of equally spaced sparse dots. These dots probably correspond to the ‘small white spots’ also described by Holm [49], which are cells more deeply situated and larger than the peripheral blastoderm nuclei. Our observations of the late blastula (Stage 4) closely match that of Holm [49] concerning the large subperipheral nuclei, which can be clearly observed with confocal microscopy.

Holm [49] summarized the studies of Faussek [60] and Schimkéwitsch [61] on the early blastoderm stage: both authors described a blastoderm composed of peripheral cells and the differentiation of a mass of cells localized in a large white spot. Faussek [60] interprets this large spot as the early genital rudiment, while Schimkéwitsch [61] terms this mass of cells the *cumulus primitivus*. Schimkéwitsch’s *cumulus primitivus* is composed of cells described to internalize into the yolk and of cells that will form the genital rudiment. A germ cell identity for this mass of cells was also proposed by Juberthie [50] in the species *Odiellus galicus* (*massa genitale*), but he did not mention cell internalization at early stages. Adjacent to this initial mass of cells, Holm [49] observed a second spot that separates from this first. He hypothesized that this second spot is the homologous structure to the true cumulus of spiders [49] (see below). According to Holm [49], the genital rudiment becomes part of a larger horseshoe-shaped field where there is a groove that is likely the remnant of the blastopore. While these cells have not been reinvestigated until the present paper, the cumulus and germ cells of arachnids are best understood in spiders.

Spider embryos have a dense spot at the center of the germ disc, termed primary thickening (synonymous with *cumulus primitivus* and *cumulus anterior*). The primary thickening is the main site of cell internalization (gastrulation) [9, 56, 57] (but see [62]). A subset of these internalized cells, termed cumulus (synonymous with *cumulus posterior* and cumulus mesenchymal cells) migrates under the ectoderm to the rim of the germ disc and establishes the dorso-ventral axis of the embryo [9, 10]. The formation of the germ cells in the spider *P. tepidariorum* is unrelated to the cumulus: clusters of germ cells are only recognizable at the germ band stage and later as paired groups of cells in the opisthosomal segments expressing high levels of germ cell markers *vasa* and *piwi* [63].

Our observations of the daddy-long-legs *P. opilio* confirm the formation of a denser spot of cells on the late blastoderm (stage 4, see Fig. 3), which is likely the site of gastrulation (i.e., blastopore). Later, we also observed, by means of time-lapse imaging, a denser spot of cells at the posterior end of the nascent germ band (Additional file 4: File S4). This mass of cells “moves” posteriorly, but this movement seems to be a consequence of the antero-posterior extension of the germband and coincides with the position where the putative primordial germ cells occur (see Juberthie [50]. While we cannot rule out the presence of a cumulus, we found no evidence of an independently moving cumulus at a stage prior to the polarized germ band (*contra* Holm, 1947). Furthermore, the germ cell marker *Popi-vasa* is expressed in the whiter cell cluster posterior of the horseshoe-shaped growth zone at stage 6 (Fig. 5), strongly suggesting their early identity as germ cells (also see below). The germ cell cluster is also characterized by larger cytoplasmic volume and loosely arranged chromatin relative to surrounding ectodermal and mesodermal cells (Fig. 5E–E′). We note that we may not yet rule out that the darker *Popi-vasa* staining at this cell cluster in early germ band stage is an artifact of the multi-layered nature of the cells, because in the spider *P. tepidariorum*, *vasa* is ubiquitously expressed in early germ disc embryos and causes the impression of higher expression at the multi-layered primitive plate [63].

Later in development, the germ cell cluster of *P. opilio* is positioned anterior to the growth zone (up until stage 9), and thereafter located on opisthosomal segment 4 (O4; stage 10). The observations suggest an anterior migration, as previously noted by Juberthie [50] in *O. gallicus*. In *P. opilio*, between stage 6 and stage 10, the germ cells migrate from the posterior to the anterior growth zone; next, they leave the growth zone to stay at the nascent O4, where they remain at least until stage 14, as also described by Moritz [52].

Outstanding questions in the early embryogenesis of Opiliones include: (1) Where and how do the early cleavages occur? (2) What is the relationship between the initial mass of cells, the blastopore, and the germ cells? (3) Does a moving organizer (cumulus) occur in Opiliones? A detailed study of the genetic markers for the cumulus [9, 10, 12, 23] and germ cells [63], in tandem with modern techniques for morphological investigation in *P*. *opilio,* offers an opportunity to provide definitive answers to these century-old questions. These future investigations will require overcoming the challenges of fixing early stages, or live imaging of the early stages with enhanced contrast and fluorescence markers through the eggshell.

### Segmentation

The sequential segmentation of the prosoma was first documented in early works from the nineteenth century (reviewed in [49], but a specific description of segment formation was only later addressed in *Phalangium opilio* [52, 58]. The segmentation of the germ band in all Opiliones investigated to date, including *P*. *opilio*, is virtually identical: it consists of the initial formation of the prosomal segments nearly simultaneously and the sequential addition of nine posterior (opisthosomal) segments and telson [52]. This mode of segmentation is conserved in arachnids, including spiders [4, 6, 7, 11, 64–66]. The initial formation of anterior segments and sequential addition of posterior segments from a growth zone is a feature also observed in Myriapoda [67] and most insects [1, 2]. At a germ band stage of the spider *P. tepidariorum*, *engrailed-1* stripes of expression in the prosoma form in a rapid sequence: the stripe in L1 appears first, followed by the stripe in the pedipalp segment and in L4, followed by the remaining stripes in the chelicera and other leg bearing segments [68]. In *P. opilio*, even though we detected the simultaneous occurrence of six stripes in stage 6 embryos and no expression in stage 5, we cannot rule out the possibility that the temporal resolution of our fixation regime was not adequate to capture the dynamic expression of *Popi-en* in the early germ band. In contrast to spiders [7, 56, 57], there are no vestiges of opisthosomal appendages throughout the development of *P*. *opilio*, consistent with the absence of appendage-like opisthosomal organs in adults of Opiliones [49, 50, 52].

The timing of limb bud elongation and opisthosomal segmentation is heterochronic in the harvestmen relative to spiders. Whereas limb buds in spiders begin to protrude when three to five opisthosomal segments have formed [56, 57], the harvestman stage with comparable limb buds coincides with just one opisthosomal segment. Similarly, by the time the harvestman embryo forms five opisthosomal segments, the tips of the legs are touching each other or crossing over the ventral midline and podomere boundaries are clearly visible. This indicates that appendage growth is greatly accelerated in harvestmen with respect to antero-posterior segmentation, in comparison to spiders. The heterochrony observed in these groups could reflect a developmental mechanism to achieve the morphogenesis of elongate appendages. Investigating this hypothesis requires further investigation of other arachnid orders with elongated appendages, such as Amblypygi (whip spiders) and Uropygi (vinegaroons).

### Neurogenesis and myogenesis

The first account of embryonic neurogenesis in Opiliones was the description in *Opilio parietinus* of “numerous small pits (…) each one surrounded by a well defined circle of cells” at a stage where the labrum is already formed [49]. Each pit is an invagination site of a group of neural precursor cells [69–72] that also occur along the ventral ectoderm. In *P. opilio*, invagination sites are first detected at stage 8, prior to the formation of the labrum and when the stomodeum begins to form. This pattern of invagination is typical of arachnids and myriapods [70, 73], in which the neural precursors invaginate as bottle-shaped cells to form the ventral nerve cord and different regions of the brain [26, 69–72]. In *Drosophila* and in spiders, neural precursors are patterned by Notch signaling, and genes in the *achaete-scute* gene complex [69–72], but these genes have not been investigated in Opiliones. A few pleiotropic genes also involved in neural development, such as *orthodenticle*, *Paired box-6*, *empty spiracles*, *dachshund*, and *Distal-less,* have been studied in *P. opilio*, and their expression patterns in the ventral ectoderm and head are comparable to the expression data in other arachnids [29, 31].

Information about embryonic myogenesis of arachnids is very sparse (reviewed by [7], and mostly undescribed in Opiliones. In general, the somatic musculature differentiates from mesodermal cells on the appendicular lobes of the paired somites along the body [7]. Winkler [58] observed paired coelomic sacs on the body segments, but did not mention further the development of the mesoderm. Moritz [52] described the development of the coxal glands from the somatic mesoderm of limb bud coeloms and depicted proximal appendicular muscle fibers. Using fluorescent antibodies, we confirmed Winkler’s observations of the prosomal appendicular lobes of the coelomic sacs and visualized putative muscle precursors in the prosomal appendages and opisthosomal segments, and the formation of the pharyngeal musculature. Considering the scarcity of data on early myogenesis in chelicerates, it would be interesting to investigate the existence of single muscle precursor cells as seen in insects and crustaceans, and test in chelicerates the utility of antibodies developed specifically to target arthropod myoblasts and muscles [74].

Phalangium opilio *as an emerging model for comparative studies of chelicerate development.*

Despite the last two decades of exciting advances in our knowledge of chelicerate development, there remains challenges to advancing the field of evolutionary developmental biology in Chelicerata for the following reasons. Firstly, classical works on the evolution of development have assumed that the development of horseshoe crabs (Xiphosura), the long-held sister group of Arachnida, reflects ancestral or plesiomorphic traits [7, 75]. Such evolutionary reconstructions require reinterpretation due to the modern understanding of the phylogenetic position of Xiphosura as nested within Arachnida, a relationship robustly recovered in recent phylogenomic studies [40, 76] (but see [77]). More generally, marine arthropod taxa tend to feature larval stages with incomplete segmentation (e.g., sea spiders; horseshoe crabs; marine crustacean groups), whereas several groups of terrestrial arthropods have convergently evolved epimorphic or hemianamorphic development. Secondly, most findings about the molecular mechanisms of development in Arachnida have only been studied in spiders. For instance, it remains unexplored if the cumulus migration mechanism of axis specification, dependent on *decapentaplegic* and *Ets4* signaling [9, 10, 23] is valid for non-spider arachnids. Thirdly, spiders and other orders of the clade Arachnopulmonata (sensu [24]) have undergone a clade-specific partial- or whole-genome duplication event [17, 19, 33, 78]. Many of these duplicated paralogs appear to have assumed new functions in development or subdivided ancestral functions [33]. Characterizing the function of single-copy orthologs in arachnids with unduplicated genomes is therefore critical to polarizing gene expression patterns and inferring ancestral states in a phylogenetic context [79]. Given our current understanding of chelicerate phylogeny, unduplicated genomes are the plesiomorphic condition of arachnids [17, 24, 33, 38]. Therefore, a broader understanding of the development and genomic evolution in chelicerates would greatly benefit from more studies in non-spider (and specifically, non-arachnopulmonate) models.

Encouragingly, non-spider models available include the horseshoe crab *Limulus polyphemus* (Xiphosura), the Arizona bark scorpion *Centruroides sculpturatus* (Scorpiones), the mites *Archegozetes longisetosus* and *Tetranychus urticae* (Acariformes), the tick *Rhipicephalus microplus* (Parasitiformes) [80], and more recently, the whip spider *Phrynus marginemaculatus* [25]. Among these species, published genomes are available for horseshoe crabs [81–85], the mite *T*. *urticae* [86], and the bark scorpion *C. sculpturatus* [17]. While all these species have revealed valuable aspects of the development of their lineages [13, 19, 87], the autapomorphic body plan of mites, and the current inaccessibility of functional approaches in scorpions (together with their phylogenetic position in the duplicated-genome clade Arachnopulmonata), are disadvantages for their use in comparative studies of chelicerate development. Gene misexpression techniques in horseshoe crabs have also not been achieved. Notably, recent advances in the deployment of CRISPR-mediated mutagenesis have been made in both mite and tick models [88, 89].

*Phalangium opilio* shares all the desirable qualities of other available non-spider models; two developmental transcriptomes and a draft genome are publicly available [29, 38]. *P. opilio* is tractable to gene expression essays by whole mount colorimetric in situ hybridization [34], HCR fluorescent in situ hybridization [38] and protein expression assays by fluorescent immunochemistry [90]. Robust protocols for embryonic RNAi are available to test gene function [28, 30, 38]. While the precise phylogenetic position of Opiliones in Arachnida remains contentious [40, 76, 77], their exclusion from Arachnopulmonata is unambiguous, as well as the unduplicated nature of their genomes [33, 38]. Harvestmen also lack the parasitic lifestyle, miniaturization, and genomic rearrangements exhibited by some mite and tick species, as well as the developmental particularities of scorpions (e.g., ovoviviparity) and horseshoe crabs (e.g., seasonal breeding; anamorphic development in postembryonic stages; threefold whole genome duplication), which incur additional challenges for developmental study. For these reasons, *Phalangium opilio* is poised to serve as a linchpin for comparative studies of chelicerate embryology and genomic evolution across Arthropoda.

## Conclusion

The embryonic staging system presented here for the emerging model organism *P. opilio* brings a fresh perspective to the exceptional classical works on the embryology of Opiliones, through the use of molecular biology and modern imaging tools. The staging provides clear landmarks to identify ontogenetic stages and has potential to facilitate collaborative research on this species. The continued research and development of tools to study the common harvestman are expected to clarify aspects of its early embryogenesis, as well as resolve which developmental mechanisms are ancestral to chelicerates and indeed all Arthropoda.

## Methods

### Embryo cultivation and fixation

Approximately 30 adult individuals of the species *Phalangium opilio* were collected during the months of May through July of 2017 in Madison, Wisconsin (USA). Animals were housed in rectangular plastic containers (25 cm × 12 cm × 12 cm) (Fig. 1C) in groups of 3–4 with at least one male and female, and kept in a room at 24ºC. Each container was provided with coconut fiber substrate, wet cotton to increase humidity, pieces of egg carton for shelter, and egg laying dishes consisting of 35 mm petri dishes with moist coconut fiber (Fig. 1C–E). Animals were fed fish flakes ad libitum, as well as freshly sacrificed *Acheta domestica*. Clutches of 15 to 200 embryos (Fig. 1D–E) laid on petri dishes were transferred to a 26ºC incubator together with large glass beakers containing water to maintain humidity. For incubation, eggs were kept in the moist coconut fiber in petri dishes, or transferred to dampened Whatman paper. For all procedures performed in the embryos, eggs were removed from the coconut fiber using blunt forceps previously touched onto a droplet of water.

For timing the embryonic development, one clutch was selected and fixed at regularly spaced time intervals. From egg laying to 5 days (120 h) after egg laying, subsets of embryos were fixed every 12 h (11 sampled intervals). From day 5 to day 13, embryos were fixed every 3 h (42 sampled intervals), and thereafter every 24 h until hatching on day 24 (seven sampled intervals). We followed hatched individuals to adulthood, recording the dates of the molts, and preserving one to two individuals at each molt in 70% ethanol. For approximate times of stages 15–19, eighteen embryos at stage 13 and 14 from a third clutch were dechorionated for 10–20 min in 50% bleach solution and photographed under 1 × phosphate buffered saline (PBS) every day until hatching.

Before fixation, embryos were rinsed in deionized water and dechorionated in 100% bleach solution for 5 min. Embryos were washed in 1 × PBS solution and fixed in the phase between 4% formaldehyde diluted in 1 × PBS supplemented with an equal volume of heptane. Fixations were carried on a shaker platform overnight (for in situ hybridization) or for a maximum of two hours (for immunohistochemistry). Fixation was stopped by 1 × PBS + 0.02% Tween-20 (Sigma-Aldrich; PBST) washes. Embryos were gradually dehydrated into 96% ethanol and stored at -20ºC.

### In situ hybridization

In situ hybridization and probe synthesis was performed according to a modified version of the protocol of Akiyama-Oda and Oda [9] for *Popi-engrailed* (*en*) and *Popi-vasa*, following [34]. Primers for *Popi-vasa* (forward: 5′-tgcctcctaaaagcgaaaga-3′; reverse: 5′-catcatccccaaaagaggaa-3′) and *Popi-en* (forward: 5′-cgtccgatttttacgttctca-3′; reverse: 5′-cgttaactcctccgttaggc-3′) were designed with T7 linkers for probe synthesis from PCR templates (5′-ggccgcgg-3′ for the forward primer, and 5′-cccggggc-3′ for the reverse). Embryos were imaged using a Nikon SMZ25 fluorescent stereomicroscope equipped with a DS-Fi2 digital color camera, driven by Nikon Elements software.

### Immunochemistry

Fluorescent immunohistochemistry was performed according to a modified version of the protocol of Akiyama-Oda & Oda [9]. Vitelline membranes were manually removed with fine forceps. Embryos were rehydrated into 1× PBS + 0.05 Triton X-100 (Sigma-Aldrich; PBS-Triton) and blocked using a 5% normal goat serum + 0.1% bovine serum in PBS-Triton. Primary antibodies consisted of Rat-Tropomyosin (Tm1) (ab50567; abcam) at 1:1500 dilution and acetylated α-tubulin (T6793; Sigma-Aldrich) at 1:500 dilution. Secondary antibodies consisted of goat α rat (Alexa Fluor 488 conjugated; Thermofisher) and goat α mouse Alexa Fluor 488 conjugated; Thermofisher) at 1:200 dilution. After staining, embryos were flat-mounted in 70% glycerol for imaging. Imaging was conducted on a Leica 710 confocal laser microscope. Maximum intensity Z-projections were generated using Fiji-ImageJ v2.0.0-rc-69/1.52n and channels were merged in Photoshop CS6 or higher by overlapping RGB color channels, or by using the screen layers function. Multi-view stacks were stitched using Zen (Zeiss).

### Time-lapse imaging

Two freshly laid egg clutches were kept for three days at 26 ºC incubator prior to preparatory procedures for time-lapse imaging. Eggs were dechorionated in 50% commercial bleach for 15 min, rinsed several times in PBS and glued to plastic petri dishes with double-sided tape. Embryos were then submerged in halocarbon 700 oil (Sigma-Aldrich) for imaging. Imaging was conducted at 20ºC–22 ºC. Images were taken every 15 min on a Nikon SMZ25. Videos of individual embryos have been cropped for clarity. For time-lapse 1 (Additional file 3: File S3), five out of twelve embryos developed normally during the experiment. For time-lapse 2 (Additional file 4: File S4), seven out of ten embryos developed normally during the experiment.

### Image editing

Some images have been cropped and/or rotated from original microscope view and were adjusted for brightness, contrast, or levels in Photoshop CS6 or higher, avoiding over-exposing pixels at any point of the micrograph. Hoechst images taken on the Nikon SMZ25 were color-adjusted in Photoshop (hue: -25) for enhanced contrast against black background. Figure plates were assembled using Illustrator CS6 or higher.

## Supplementary Information

**Additional file 1: File S1**. Oviposition of egg clutch. This video shows a female with protruded ovipositor laying one egg in a clutch deposited in wet cotton.

**Additional file 2: File S2**. Overview of *P. opilio* development, presenting main features of each stage and approximate timing. Greyscale images were stained with the nuclear marker Hoechst.

**Additional file 3: File S3**. Time-lapse imaging of embryos of the same clutch 3 days after egg laying. Imaging under oil. Videos have been cropped from the same microscope view. Left: Ventral view; head is to the right. Center: Lateral view; head is to the right. Right: Ventral view; head is to the left. Pictures have been taken every 15 min.

**Additional file 4: File S4**. Time-lapse imaging of embryos of the same clutch (but different from S1–3) 3 days after egg laying. Imaging under oil. Videos have been cropped from the same microscope view. Left: Dorsal view; head is to the right. Yellow arrowhead at 100.5 h marks the denser spot of cells. Center: Dorsal view, head is facing down. Right: lateral-ventral view; head is facing down. Pictures have been taken every 15 min.

**Additional file 5: File S5**. Hatching of *Phalangium opilio* into the postembryo. Each frame corresponds to a 10s interval.

**Additional file 6: File S6**. First molt of *Phalangium opilio*, from postembryo to the first instar. Each frame corresponds to a 10s interval.

## Data Availability

All data generated or analysed during this study are included in this published article [and its Additional files].
