# Supplementary figures and images for: Eggs to long-legs: embryonic staging of the harvestman Phalangium opilio (Opiliones), an emerging model arachnid
Source: Front Zool. 2022 Mar 4;19:11. doi: 10.1186/s12983-022-00454-z (PMC8896363; doi:10.1186/s12983-022-00454-z)

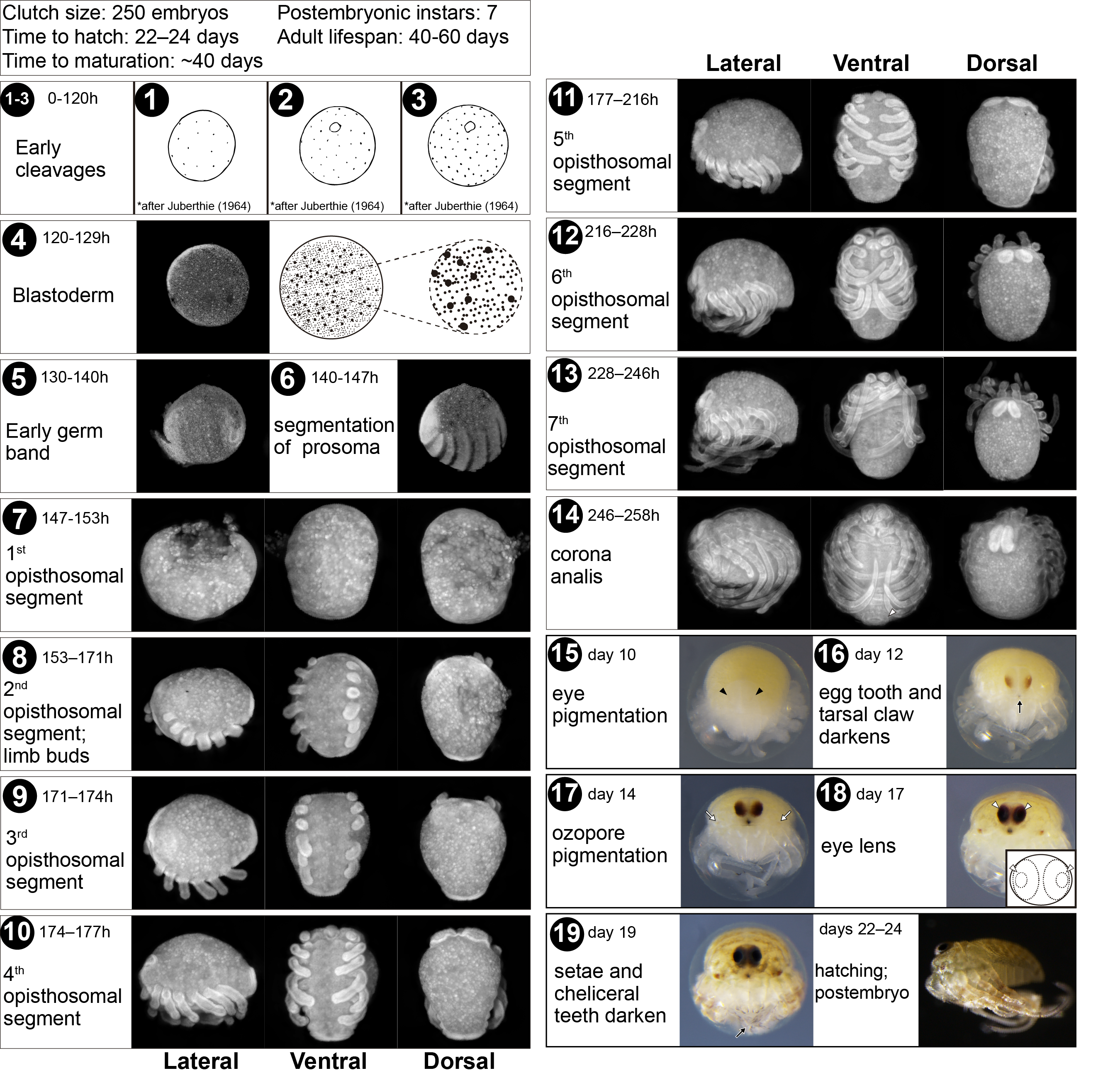

Supplement: Supplementary file 2 — Additional file 2: File S2. Overview of P. opilio development, presenting main features of each stage and approximate timing. Greyscale images were stained with the nuclear marker Hoechst. [file 12983_2022_454_MOESM2_ESM.png]
